# Rurally rooted cross-border migrant workers from Myanmar, Covid-19, and agrarian movements

**DOI:** 10.1007/s10460-021-10262-6

**Published:** 2021-09-03

**Authors:** Saturnino M. Borras, Jennifer C. Franco, Doi Ra, Tom Kramer, Mi Kamoon, Phwe Phyu, Khu Khu Ju, Pietje Vervest, Mary Oo, Kyar Yin Shell, Thu Maung Soe, Ze Dau, Mi Phyu, Mi Saryar Poine, Mi Pakao Jumper, Nai Sawor Mon, Khun Oo, Kyaw Thu, Nwet Kay Khine, Tun Tun Naing, Nila Papa, Lway Htwe Htwe, Lway Hlar Reang, Lway Poe Jay, Naw Seng Jai, Yunan Xu, Chunyu Wang, Jingzhong Ye

**Affiliations:** 1 International Institute of Social Studies (ISS) of Erasmus University Rotterdam, Korternaerkade 12, 2518 AX The Hague, The Netherlands; 2 Transnational Institute (TNI), De Wittenstraat 25, 1052 AK Amsterdam, The Netherlands; 3 Justice Society, Northern Shan State, Myanmar; 4 Lahu Development Network, Eastern Shan State, Myanmar; 5 Metta Development Foundation, Pegu, Myanmar; 6 Mon Area Community Development Organization, Mon State, Myanmar; 7 Mon Women Organization, Mon State, Myanmar; 8 Mon Youth Progressive Organization, Mon State, Myanmar; 9 Mon Region Land Policy Affair Committee, Mon State, Myanmar; 10 Pa-O Youth Organization, Southern Shan State, Myanmar; 11 Paung Ku, Yangon, Myanmar; 12 Ta’ang Students and Youth Organization, Northern Shan State, Myanmar; 13 Tai Youth Network, Northern Shan State, Myanmar; 14 College of Humanities and Development Studies (COHD) of China Agricultural University, No. 2 West Yuanmingyuan Road, Haidan District, Beijing, 100193 People’s Republic of China

**Keywords:** Migrant workers, Farmworkers, Covid-19 pandemic, Myanmar, Food sovereignty

## Abstract

This paper examines the situation of rurally rooted cross-border migrant workers from Myanmar during the Covid-19 pandemic. It looks at the circumstances of the migrants prior to the global health emergency, before exploring possibilities for a post-pandemic future for this stratum of the working people by raising critical questions addressed to agrarian movements. It does this by focusing on the nature and dynamics of the nexus of land and labour in the context of production and social reproduction, a view that in the context of rurally rooted cross-border migrant workers necessarily requires interrelated perspectives on labour, agrarian, and food justice struggles. This requires a rethinking of the role of land, not as a factor in either production or social reproduction, but as a central component in both spheres simultaneously. The question is not ‘whether’ it is necessary and desirable to forge multi-class coalitions and struggles against external capital, while not losing sight of the exploitative relations within rural communities and the household; rather, the question is ‘how’ to achieve this. It will require a messy recursive process, going back and forth between theoretical exploration and practical politics.

## Introduction

*We at ECVC cannot accept that maintaining food production continues to be done at the expense of the health, rights and dignity of rural and migrant workers.*

European Coordination Via Campesina (ECVC) 30 April 2020 letter to the European Commission & European Parliament.^1^

*There is hope now that the wheels of societal change are beginning to turn faster during this pandemic. It is indeed time to transform. The rights of people, dignity, and solidarity, not profits, should be the foundation of the new society. Food sovereignty is the right and just path.*

La Via Campesina, 13 August 2020.^2^

A radical transformation of the global agro-food system will be a key component in the kind of systemic change that is required for a positive post-pandemic future.^3^ The two statements above—by European Coordination Via Campesina (ECVC) underscoring the health, rights, and dignity of rural and migrant workers, and by La Via Campesina reiterating that food sovereignty is the right and just path into the future—shine a light on the two clusters of objectively linked issues of workers and peasants as having a key role in a post-pandemic world. This is a positive step in terms of academic research and political activism because rural workers, including migrant workers, and their issues around jobs, wages, and working conditions are not always viewed from the perspective of the ‘peasant way’ or ‘people of the land’ in discussions on food sovereignty or agroecology, despite the centrality of labour in such narratives and practice. Farmers who hire workers, and workers who sell their labour power to farmers (to varying degrees), may share a common vision of a just transition into a positive future—e.g., ‘agricultural sector with more people in it’ (in relation to agrarian movements’ critique of industrial agriculture as ‘agriculture without people’) but this does not easily or automatically resolve conflicting class interests and contradictory political impulses. What role the global agro-food sector could play in efforts to construct post-pandemic alternatives, such as a (global) ‘green new deal’ (Patel and Goodman 2020; Selwyn 2021), is in part dependent on how, and how well, a range of fundamental issues for peasants^4^*and* workers are addressed. We aspire to make a modest contribution to this conversation by focusing on a subgroup of the peasantry, or indeed the working class, namely, cross-border migrant workers in the agro-food sector who are rooted in rural areas.

The precarious lives of migrant workers in the global economic system have been underscored during the pandemic. Many were trapped in their livelihood sites but without work and means to feed themselves; some were stranded while trying to return home; still others got stuck somewhere en route back to their work sites (Rao et al. 2020). Migrant worker’s dormitories became sites of concentrated and rapid Covid-19 infection, given the cramped living quarters (e.g., Koh 2020; Suhardiman et al. 2021; Xiuhtecutli and Shattuck 2021). ‘No work, no pay’ is the general condition of work for this social group. It is the section of the working class that was in the most precarious situation before and during the pandemic, and is captured, to varying extents, by some of the relevant concepts such as ‘precariat’ (Standing 2014), ‘footloose’ labour (Breman 1996), ‘working people’ (Shivji 2017), and ‘classes of labour’ (Bernstein 2006). The uneven impact of the pandemic has exposed pre-existing class-based, nationality-based, racialized, and gendered inequities, as well as the structural and institutional character of the global food system (Clapp and Moseley 2020; Guido et al. 2020; Xiuhtecutli and Shattuck 2021).

Within the broad grouping of migrant workers, a subset that has been exposed during the pandemic is the cross-border migrant workers in the agro-food system. These are migrant workers in the farms (e.g., strawberry pickers, sugarcane cutters), meat-packing factories, large-scale industrial pig and chicken farms, food processing and distribution centres, restaurants, large-scale agribusiness plantations (e.g., oil palm), and so on. They are found in both the North and the South. There are several factors that triggered their being thrust front and centre of media and public attention during the pandemic. These included, first, the breakdown of the global food supply chain, especially during the early phase of the pandemic, caused in part by the inability of migrant workers to be where they should be and do what they should do at the required time of commodity production, processing, and distribution (Bello 2020; Clapp and Moseley 2020; Klassen and Murphy 2020; van der Ploeg 2020); second, localized outbreaks where migrant workers were concentrated, whether at a work place, at their dormitories, or in informal urban settlements (Corburn et al. 2020); finally, distressing scenes of families of migrant workers who had lost jobs and who struggled to survive, alongside parallel scenes of migrant workers who continued to work, without being able to observe standard medical protocols on public healthy safety (Accorsi et al. 2020).

Two clusters of academic research and political activist advocacy have emerged in terms of exploring ideas of the place and role of migrant workers in the agro-food system during the pandemic and in a post-pandemic new normal. The first cluster is focused on labour issues, mainly advocacies for labour justice, health justice, and cross-cutting social justice issues on race, ethnicity, and gender (e.g., Haley et al. 2020; Marcom et al. 2020; Xiuhtecutli and Shattuck 2021). The second cluster is focused on agro-food systems. Food sovereignty, anchored in agroecology, remains the dominant alternative scenario in this regard, as in the rallying call by La Via Campesia (Altieri and Nicholls 2020; Montenegro de Wit 2021; van der Ploeg 2020). We build from these two clusters, and explore a middle ground which combines elements of both.

Our paper is located in the broad discussion above, but is narrowly focused on the subset of cross-border migrant workers in the agro-food sector who are rooted in rural areas. This smaller subset is defined by a number of characteristics: first, we are talking about migrant workers who work outside of their home country. Second, they work in the agro-food sector, which is more encompassing than the subgroup of ‘migrant farmworkers’, and includes workers in farm production, service, trading, processing, distribution in the food sector (e.g., sugarcane cutters, haulers) and non-food sector (e.g., rubber tappers, plantation maintenance crew), agro-food processing, retail factories, shops, grocery stores, restaurants, and so on. Third, most jobs in this sector are seasonal, but this does not necessarily mean that migrant workers periodically return to their home country; some of them hop from one form of seasonal wage work to another in the same country of work. For example, a Myanmar migrant worker in Yunnan (China) harvests macadamia in September–October, cuts sugarcane from November to early May, and may then go back to Myanmar in mid-May at the onset of the rainy season to prepare land on their own farm, although some simply continue to look for other seasonal wage work in Yunnan between the end of the sugarcane cutting season and the start of the next macadamia harvest. Fourth, by ‘rural-rootedness’ we mean that they have not completely cut their ties to their rural roots in their country of origin when viewed across time—past, present and future. Finally, they produce commodities and reproduce themselves simultaneously in the same logic and process of global capitalism, so that (following Bhattacharya 2017; O’Laughlin 1977; Shah and Lerche 2020) we take a unitary view of production and social reproduction.^5^

Our interest is in the nexus between land and labour, with an assumption that, for rurally rooted cross-border migrant workers, land is crucial to social reproduction, broadly cast. We take our signal from Shah and Lerche (2020, p. 720) who argue that in migrant labour studies there is less attention given to the issue of “how appropriation of surplus value from labour occurs both at the site of production as well as through labours’ social reproduction… situated not only in the place of migration but, importantly, also in migrants’ home areas”. They argue that in the home areas, we also find “productive activities, challenging a production–reproduction divide” (ibid.). More broadly, we build on Fraser:

Non-waged social-reproductive activity is necessary to the existence of waged work, the accumulation of surplus value and the functioning of capitalism as such. None of those things could exist in the absence of housework, child-rearing, schooling, affective care and a host of other activities which serve to produce new generations of workers and replenish existing ones, as well as to maintain social bonds and shared understandings. Social reproduction is an indispensable background condition^6^ for the possibility of economic production in a capitalist society. (Fraser 2016, pp. 101–102)

For us, most of these social reproductive activities cannot take place without a range of access to land by working people. We build on and contribute to the discussions by Shah and Lerche, Fraser, Bhattacharya, and others by taking a much narrower unit of inquiry—rurally rooted cross-border migrant workers in the agro-food sector—focusing on the role played by social dynamics of land and labour regimes. But even in this narrower unit of inquiry, the sites of production and social reproduction are diverse, multiple, and often fluid, akin to a web. These sites are connected by multiple corridors of largely informal, and even illegal, terrain ruled by capitalists, petty bureaucrats, police, labour traders, and lumpen elements.

Among the social groupings within the universe of migrant wage workers, this subgroup might be more difficult to account for and track because the extent and degree of fluidity, informality and/or illegality, compared to other subgroups, are likely to be greater. Historically, there have been recognized corridors for this type of migrant subgroup; some are long gone, others have persisted. The second half of the nineteenth century saw waves of migrant workers (Chinese, Japanese, Filipino, Indian, and domestic migrants) in the agro-food sector who built the modern agriculture of California; this is an example of an historical moment that has passed, although its structural and institutional legacies linger (McWilliams 2000 [orig. 1935]; Mitchell 2012; Street 2004). California also saw multiple waves of Mexican migrant workers, from the early twentieth century to the period of mass expulsions during the Great Depression (Guerin-Gonzales 1994; McWilliams 2000 [orig. 1935]); again, from the 1940s onwards as a result of the Bracero Program; and right up to the tumultuous Trump era (Holmes 2013; Mitchell 2012; Ngai 2014 [orig. 2004]; Minkoff-Zern 2019; Xiuhtecutli and Shattuck 2021). Most of the migrant workers involved in these historical processes in California were rooted in the rural societies of their countries of origin (Guerin-Gonzales 1994).

This broader and longer perspective is needed for understanding the conjunctural situation of rurally rooted cross-border migrant workers in the agro-food sector in the era of Covid-19 and beyond. We need such an analytical lens in studying the impact of the pandemic and government responses to it on this particular social group in relatively newer corridors, including Myanmar–China/Thailand/Malaysia (Deshingkar et al. 2019; Suhardiman et al. 2021), Vietnam–China, Indonesia–Malaysia, Bangladesh/India–Malaysia, Nicaragua–Costa Rica, Venezuela–Colombia/Chile, Bolivia–Argentina, Zimbabwe–South Africa, Morocco-Europe (Corrado et al. 2016), and Mexico/Central America–North America. It is important to look into this category not only because it comprises a large and growing social group, but also because the academic and political resonance may be wider than previously realized.

Our main arguments are as follows. First, the pandemic has exposed latent issues confronting a specific social subgroup of the working people, i.e., rurally rooted cross-border migrant workers, related to land and labour, as well as production and social reproduction, that have hitherto not been fully explored and understood, academically and politically. This rests partly on a key starting point in this study that a significant section of the world’s peasants occasionally or regularly hires out labour themselves (both as a matter of degree), conforming less to the conventional peasant or farmer label, but rather approximating the description of ‘working people’ (Shivji 2017) or ‘classes of labour’ (Bernstein 2006). Second, in rural development-related literature and narratives, the dominant interpretation of the meaning of land is situated in economic production, usually understood as farmland. For the social subgroup of the working people that we study, the meaning and importance of land have to be recentred to the inseparable economic production *and* social reproduction. For the latter it implies a range of access: from a freehold plot or space in the village commons for housing, to access to a community forest, to access to a public space for school, clinic, and socialization purposes. Third, a range of access to a range of land that in turn conditions how wage labour is activated and deployed by working people is shaped and mediated not by either market relations only *or* extra-economic coercion, *but by both*. Violence, especially in the case of Myanmar, is key in shaping and mediating both market relations and extra-economic coercion. Finally, contemporary anticapitalist struggles, especially those concerning agrarian, food, environmental and labour justice may have to introduce greater degree of nuance about questions of subjective forces, strategy, demand-making dynamics, and alternatives—at least in relation to the rurally rooted cross-border migrant workers. These arguments and their academic and political implications were urgent and important in the situation before the pandemic and the February 2021 military coup in Myanmar; these have become exponentially even more so given the pandemic and the military coup. Their resonance is far beyond Myanmar, or so we hope.

## Research methods

**Fig. 1:**
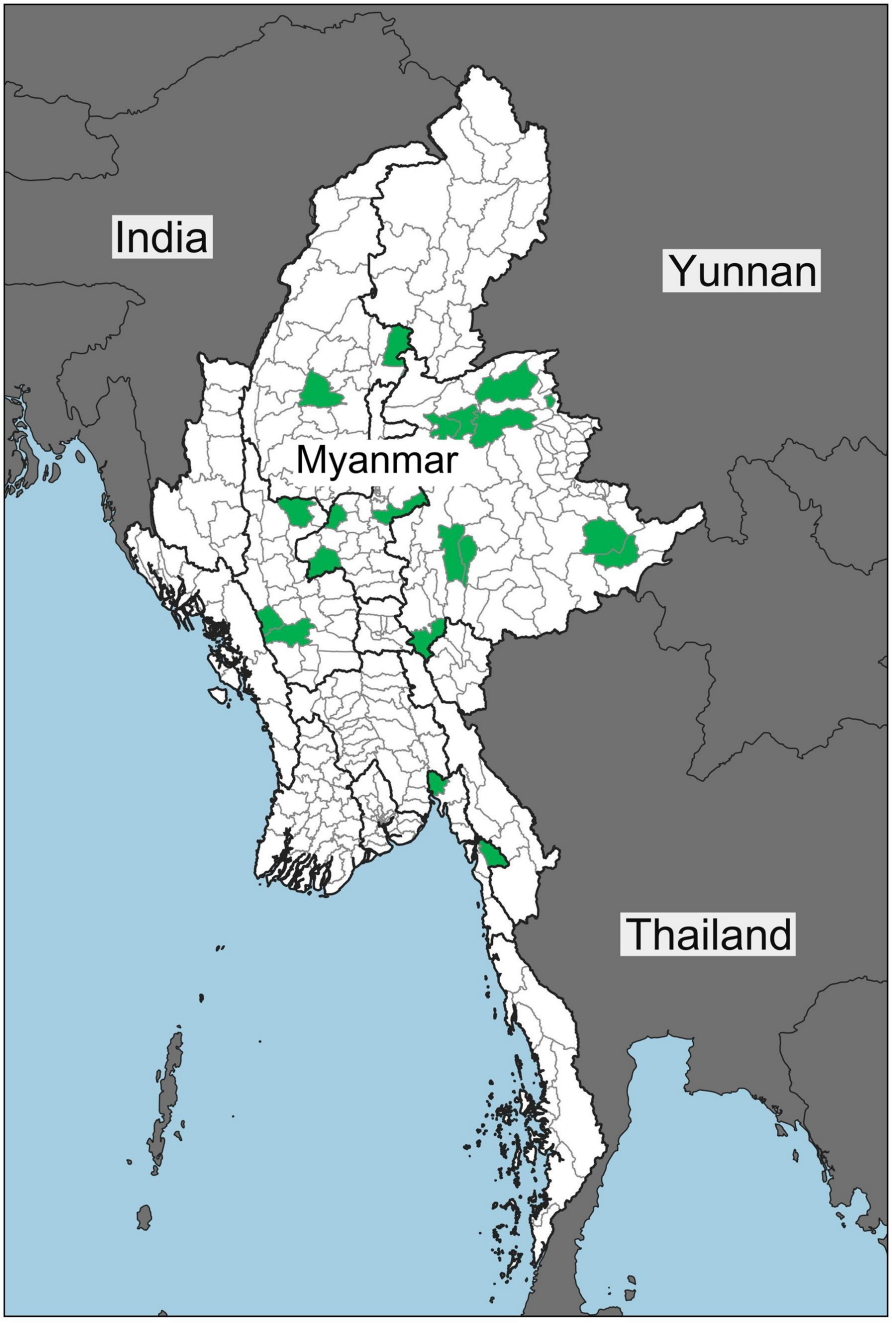
Map of Myanmar in regional perspective (shaded parts in the Myanmar map are the regions from where the migrant workers we interviewed come from) (Original map was obtained from www.themimu.info and adapted by Aung Thu. It is an open source website for the development sector)

In August–September 2019, we interviewed 16 migrant workers from Myanmar’s Dry Zone (Magwe, Mandalay, Sagaing) who work as sugarcane cutters in China. After the pandemic erupted, we decided in April 2020 to carry out interviews by phone or by Facebook Messenger and, whenever possible, by face-to-face interview observing the government-stipulated safety measures amid the pandemic. Our initial focus on the Dry Zone expanded to include migrant workers from Shan and Mon States. The decision to include Shan and Mon States was based on two factors: the presence of existing research collaborators, and the significance of migrant wage work in these two states.^7^ The research methods used were purposive sampling and the snowball technique. All the co-authors of this paper have long-standing networks within the communities. We relied on these pre-existing local networks to make the first contact and to build an initial pool of interviewees. All interviews were conducted in the local languages of the interviewees. We anonymized interviewees’ names, and withheld the names of their villages to protect their privacy and ensure their safety and security. Apart from the 16 migrant workers interviewed in August–September, 2019, the remaining interviews were carried out in May–August 2020, involving 120 migrant workers. “Annex 1” presents the profiles of the 136 interviewees.^8^ Figure 1 present Myanmar geographic location in regional perspective.

## The rise of rurally rooted cross-border migrant workers

Below are narratives from three different interviewees that demonstrate the links between productive and social reproductive activities, as well as the past, present and future in the lives and livelihoods of migrant workers:

We have 5 acres of farmland that I inherited from my parents, which used to be bigger than that, but in the 1990s the military grabbed lands from the villagers including from my parents. My wife and I work in the farm and cultivate crops, including beans and rice. But most years we could not make any profit from farming. In a good year, the most profit for the entire year is equivalent to one month’s wages from working in China. So, I went to China in 2018. Sugarcane cutting is from November to May. Afterwards, I come home and work in our farmland, the produce of which is just enough for family consumption. I also work as a palm tree climber in nearby communities in February to July. Then in November, I go again to China. The majority of the households in my village have members who regularly go to China to work, with only the older people and children left behind. Many bring their babies and very small children with them to China. Last time, I brought with me my 15-year-old son. He is strong. He can cut up to 200 bundles of sugarcane in one day! I can only cut an average of 80 bundles a day.^9^

I had to quit school in my seventh grade to help out my parents by looking for wood for fuel, rubber tapping and selling tamarind leaves. Even though they have a rubber farm in the village, it was not enough to feed the family. So, I went to Thailand and worked different jobs. After I married, my husband and I worked at a rubber plantation. I got very sick while working at the rubber plantation and came back to my home village and went to Yangon to seek healthcare. When I got better, I went back to Thailand. When I was pregnant and had to deliver the baby, I was alone travelling back to Myanmar to deliver the baby. Now our daughter is 2 years old. During the pandemic, we just stayed within the compound in Thailand. Before, we had planned to send our daughter to our home village, but because the roads were blocked, we could not do that anymore. The weather has now changed to rainy season, so I am worried my daughter might contract dengue or malaria. And I feel it unsafe to leave her alone while I’m doing rubber tapping. So, when I go to rubber tapping work, I have to take her to the rubber plantation. I position the baby within sight, with a companion dog, so that I can work.^10^

I have been to Thailand as a migrant worker three times already. First time was when I was 12 years old. I worked for one year scooping charcoal from a big hole in the ground where the charcoal is burned and made. It’s very hard on my back. It takes me three to four days to finish scooping charcoal in one hole. I was paid a very small amount of money. Then I went back to Myanmar. When I was 17 years old, I went back to Thailand and worked at a construction site. Then I went back to Myanmar. In January 2020 I went back to Thailand, until April 2020. Then I went back to my village because of the pandemic. I don’t want to go back to Thailand. Maybe I used to scoop charcoal too much for too long because now my back is severely painful all the time.^11^

These interview quotations are a tale of how the logic of capital triggers the process in which commodities are produced and life is reproduced simultaneously in diverse and multiple sites of the co-constitutive productive and social reproductive spheres across nationally bounded societies. Productive and reproductive, waged and unwaged work are simultaneously done in China and in Myanmar, in the homes and communities, and at the migrant work sites. Their livelihood earnings at home are not sufficient for them to survive and have to be augmented by their wage work within their local community and/or abroad. Or, seen from the other end of the corridor, their migrant wage work earnings are not sufficient, and thus have to be supplemented by working the land and/or selling labour power in their home community. This difficult condition was part of their everyday life before the pandemic; it became even more challenging during the pandemic.

The national development strategy of the Myanmar government depends on two key pillars, namely, extraction of natural resources (oil and gas, and others) and export of labour, with a relatively small industrial sector centered around the garment industry. According to the World Bank (2019, p. 16), “Myanmar officials stated… that 5 million Myanmar nationals are working overseas with about 3 million in Thailand, mostly in manual labor”, and a significant part of migrant labour is located in the grey area between what is legal and what is not in terms of labour laws in the countries of work. Partly, because of the informality of migrant work, it is likely that estimates of the number of migrant workers and their contribution to the economy are conservative. According to the International Organization on Migration (IOM): “While official estimates are that Myanmar only received $118 million USD in remittances in 2015, the then Ministry of Labour, Employment and Social Security estimated that remittances could be as high as $8 billion USD”.^12^ The latter figure would represent about 11% of the country’s GDP that year.^13^ Regardless of the exact value, between a low of US$2.8 billion (KNOMAD 2020) and a high of US$8 billion annual remittances, what these data show is that the contribution of migrant workers to the national economy is significant.

The national strategy for capitalist accumulation is made possible in part by making sure that the rural areas and agricultural sector shoulder some of the requisites of such a process. This is in the form of maintaining affordable food for the working people, and by letting the latter take care of the bulk of social reproduction cost by themselves largely by relying on access to rural spaces and resources. In 2020, 70% of the 53.7 million population of Myanmar were living in rural areas. The development of the agricultural sector has been uneven overtime and across subsectors. There has been some modest growth in terms of exports of rice and beans. The more significant expansion has been in corn, sugarcane, banana, rubber and oil palm production—and these subsectors are all associated with the land rush that have started around 2000 and peaked in 2010–2012.^14^ The agriculture sector contributes enormously to the national economy. It gives far more than it receives from the government: in 2017, the agriculture sector accounted for 23.7% of GDP and provided some kind of livelihood to 50% of those reported to be ‘employed’ in the sector, while it received a share of just 3.8% of total government expenditure that year (World Bank 2018, p. 46). From 2009 to 2017, “agricultural public expenditures in Myanmar averaged 1% of GDP and 6.5% of the Union budget” (World Bank 2017, p. 21). Overall, the country’s GDP has been growing at an impressive annual rate, although the growth rate of value added in agriculture is not as eye-catching. This follows a familiar pattern of neoliberal development growth rates more generally: continued economic growth rates that do not trickle down to the vast majority. Given this economic structure, the rural communities contribute to the national economy in part by providing cheap labour to the emerging top sector, which is the service sector, and by exporting cheap labour. In this way the rural sector reduces national unemployment, or conceals the extent of real under- and unemployment because 50% of those deemed employed in agriculture are poor peasants who could barely survive from farming and are unable to find regular waged work. “Poverty is more prevalent in rural areas. The number of poor people is also 6.7 times higher in rural areas than urban areas, and those residing in rural areas make up an overwhelming majority (87%) of the nation’s poor” (Myanmar Union Government 2019, p. xi).

The level of land and labour productivity in Myanmar is generally low, while at the same time a class of petty and big capital has emerged in the rural areas (traders, agribusiness companies, real estate brokers, military institutions) that is influential enough to contribute to shaping state policies toward agriculture and capital accumulation in favour of exclusionary, extractive approaches such as big plantations, or outgrowership (Myanmar Union Government 2019; Vicol and Pritchard 2020). While the production of impoverished rural working people are the outcomes of both market relations and extra-economic coercion, the latter, often in the form of brute force and violent conflict are quite pervasive in Myanmar society and have played a key role in the making of Myanmar rural ‘working people’ (along the definition by Shivji and Bernstein). The rise of a handful powerful economic and political elite who are, one way or another, linked to the military—emerging merchant capital, landlords, agribusiness, finance capitalist, bureaucrat capitalists, or what ordinary Myanmar people refer to, collectively and pejoratively, as ‘crony capitalists’ has also been noticeable during the past couple of decades.

It is the working people’s insertion into this social structure that largely shapes the character and degree of their vulnerability. There is a tendency to make the causes of poverty sound natural: ‘no rain and so people starved’.^15^ Yet we see prosperous capitalist farm enterprises and ranching activities in the same region at the same time that we see poverty. In fact, the same villagers who appear to be resigned about the natural agroecological conditions that they cited during our interview as the cause of their poverty are leasing lands or are working for emerging thriving capitalist farms financed by outside merchant capital, mostly from China. These conditions in production and reproduction in rural Myanmar drive peasants, agro-pastoralists, artisanal fishers, and landless labourers, especially the young and middle-aged men and women, to take up a variety of productive and reproductive activities, including on-farm and off-farm wage work within and outside Myanmar. Not all villagers in Myanmar’s countryside seek wage work, domestically or abroad. A 45-year-old man from the Dry Zone told us:

I bought ten goats when I got back from China in May [2019]. At least when the situation is bad, we can sell them one at a time. The income from China is good. But it is just enough for our subsistence. There are 180 households in my village. Almost all of them have members who go to China to work, except for those very few households who have a shop or big farm or a big animal herd with capital.^16^

While migrant workers we have interviewed emphasized the need to remain inserted into the migrant labour regime, it does not mean that their productive and social reproductive activities in the home communities are less important. These include their own farming (whether aimed at subsistence or accumulation), housing needs, access to social services (for children’s schooling, convalescence needs, health clinics, biological reproduction needs, etc.), kin networks (for childcare, etc.), and, ultimately, provision for retirement. Indeed, it is not a matter of which is more important: as stated at the beginning of this paper, our conceptual reference point is that we see productive and social reproductive spheres as co-constitutive. As we framed in the beginning, and of particular relevance is our starting assumption that access to land cannot be reduced to the issue of access to a farm plot; rather, it has to be seen as access to a range of land and resources, including public spaces such as parks for children’s play or access to water sources for drinking, cooking, and washing. Thus, the availability of migrant work on the one hand and the availability of land for housing back home weigh equally heavily: they are inseparable issues for the subset of migrant workers in our study. In the words of a 22-year-old Shan man:

Previously there was no fighting in our community, so each family had access to land for farming. But later the army came, and fighting with ethnic armed organization started. That’s why many in our community became migrant workers in China because we lost our livelihood in our village. Farmers who were able to work for a good living before, are now struggling very hard for a living; some have difficulty finding ways to even eat regularly, and some have become homeless. Our village has around 40 households, only 15 households have a house in the village centre, and most of the families who don’t have house plots rent spaces from villagers, or stay with relatives, who have houses. Most of the families who don’t have a house have sent their children to lower Myanmar for education. In these cases, we often see those children facing too many difficulties and multiple challenges. After losing access to farmland and house lots, we have seen many negative consequences in the lives and livelihoods of the affected families.

It is important to point out, however, that throughout the research process for this study, when we ask interviewees whether they have land, and whether they want or need land, the general understanding of ‘land’ is ‘farmland’ (only). A weakness in our research process was that we did not specifically emphasize the matter of ‘a range of access to a range of land’ as the more appropriate framework to understand land in relation to our unitary perspective on productive and social reproductive activities. This means land as ‘farmland and beyond’: from farmland to community forest, from a space for housing (whether freehold or a space in the village commons) to community space for school or clinic or simply for general socializing. Our tabulated dataset on land access, therefore, should be read with its given weakness.

There is a pattern among the 136 migrant workers we interviewed in terms of the situation they face in trying to make a living in their home villages. Tables 1 and 2 capture some highlights. First, 63% of those we interviewed have farmlands, but that their farmland is either too small or of poor quality, or both. These data challenge a popular assumption that giving farmland to rural villagers, or indeed giving farmland back to Internally Displaced Persons (IDPs), is sort of a guarantee to prevent impoverishment in the rural areas which will, in turn, reduce the appeal of migrant work. It reminds us of our conceptual argument in the beginning that farmland in economic production sense alone is a necessary but not sufficient condition for a robust livelihood; *a range of access to a range of land* in productive *and* social reproductive activities is key. Second, 95% of those who said they have farmland, also said that the income generated from their farmland is not sufficient.^17^ Third, 28% do not have farmland.

**Table 1:** Land access and production-related issues for migrant workers interviewed

Issue	Migrants from Dry Zone N = 39	Migrants from Shan State N = 74	Migrants from Mon State N = 23	Total N = 136
Access to farmland				
No farmland	12	19	7	38
% of the region	30.8	25.7	18.4	27.9
With farmland	18	47	13	78
% of the region	46.2	63.5	56.5	57.4
No response	9	8	3	20
% of the region	23.1	10.8	13.0	14.7
Access to wage work				
Hiring out labour: wage working in and near home communities, but not sufficient income	19	42	15	76
% of the region	48.7	56.8	65.2	55.9

**Table 2:** Production-related issues for the 78 migrant workers who have farmland at home in Myanmar

Issue	Migrants from Dry Zone N = 18	Migrants from Shan State N = 47	Migrants from Mon State N = 13	Total N = 78
With farmland but too small and/or of poor quality	7	34	8	49
% of those have land access in the region	38.9	72.3	61.5	62.8
Farmland, livestock production for market: not sufficient income	18	44	12	74
% of those have land access in the region	100.0	93.6	92.3	94.9
Market price for produce as a problem	9	19	1	29
% of those have land access in the region	50.0	40.4	7.7	37.2
High cost of farm inputs as a problem	5	17	2	24
% of those have land access in the region	27.8	36.2	15.4	30.8
Problem of weather and pests (drought, flooding, etc.)	7	1	0	8
% of those have land access in the region	38.9	2.1	0.0	10.3
No irrigation	6	0	0	6
% of those have land access in the region	33.3	0.0	0.0	7.7
Hiring in labour to work their farmland, livestock (while in migrant work)	6	0	0	6
% of those have land access in the region	33.3	0.0	0.0	7.7

Fourth, 56% relied on wage work in or near their home villages, before or in between periods of migrant wage work. This was the case across the categories of those who have and do not have farmland. They see the importance of having access to farmland as one of the necessary conditions for sustaining their households that stay behind in the home villages: to maintain farm production (largely, though not solely, for daily consumption), to maintain their houses (if they live in their own house), to have access to community forest or grazing area, a place for convalescence in times of illness and for eventual retirement when they can no longer physically continue doing migrant wage work, and to provide a material basis for their sense of belonging to a community that is critical to many other aspects of social reproduction. Logically, and as hinted in many of the interview materials we have for this study, the importance of having a range of access to a range of land, as described above, is not only relevant for those who have some farmland back home; it is equally important for those who do not have farmland at all. In short, a range of access to a range of land back home is key to keeping their rootedness to their community, now and in the future, where social care can be coordinated and carried out: childcare, schooling of children, care for elderly household members, and indeed as the locus for their own healthcare and retirement.

## The Covid-19 crisis: neither the first nor the last crisis for migrant workers

Taking the decision whether to go back home because of the pandemic was not easy for many, especially if it meant losing a job—or losing the wages they had already earned. Our research suggests that, for migrant work in southern China, it is the norm that the China-based labour broker (the ‘boss’) is the direct employer of the worker, and not the farm or factory owners, at least in the agro-food sector. The boss pays the workers’ wages only partially, with full payment made according to an agreed timetable, usually at the end of the season’s work cycle. When migrant workers decided to return home during the pandemic, most of them were not paid the full wages they had already worked for. These losses, combined with a pre-existing crisis of production and social reproduction in the home village, are among the most significant negative socio-economic impacts of the pandemic on the rurally rooted cross-border migrant workers.

**Fig. 2:**
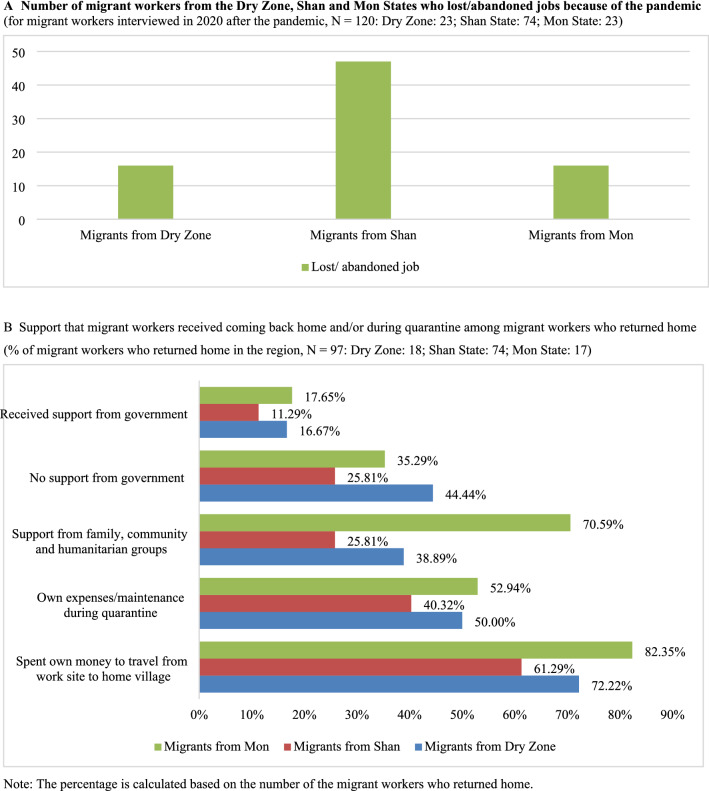
Migrant workers and employment

In the 120 interviews we conducted among migrant workers *during* the pandemic, two out of every three migrant workers lost jobs or abandoned jobs because of the pandemic, either returning to Myanmar, or staying in the countries of their work (see Fig. 2, Panel A). These figures are telling, but not surprising. Panel B of Fig. 2 shows what support, if any, the migrant workers received. The category “spent own money to travel from work site to home village” has high percentages of 61%, 72%, 82% for Shan, Dry Zone and Mon migrants, respectively. For those who “received support from government to come home and/or during quarantine” the percentages are at a low: 11%, 17%, and 16% for Shan, Mon and Dry Zone migrants, respectively.

## Informality: facilitating labour flows, weakening labour struggles

I worked in a watermelon farm in China, together with 15 other people from the Dry Zone. We crossed the border illegally; we rode three different cars to get to the peak of the mountain along the border, crossed the mountain border, and then we rode another car that brought us to the farm. We were accompanied by a labour contractor who is also from the Dry Zone. We were given free accommodation and food. One hall for all men, and another room for women. We sprayed fertilizer and pesticide, and we covered watermelon with foam. We worked seven days a week. If you get sick, you have to pay for your costs, and you will have no wage for the days you were ill and could not work. We were given only half of our wages with the understanding that the full payment would be given when we go home to Myanmar on the agreed date. When the police came, the boss would warn us, and we had to hide. The watermelon field has a depression; if the police were coming, we had to run into the depression, cover ourselves with leaves and branches and hide there until the police had left. After working there for two months, we were unhappy because the work was hard, the pay was too little and the boss was not very nice. One day, we ran away. We pre-arranged with a Shan Chinese truck driver to bring us to a particular point. Then we climbed a mountain for one and half hours. We had to crawl under a barbed wire fence to cross into the Myanmar side. When we got home, we had no more money. For the two months we worked, we only got the wages for one month. The work in China was hard, but it’s a regular job. I want to go back there.^18^

In our case study, it is not just any type of labour that is being sought: what is needed is manual labour for jobs that the domestic workforce does not want to take, because these are among the lowest paid or the most precarious jobs in terms of period of work or certainty of work.^19^ Many such jobs are in secondary and smaller cities or small and medium towns, or deep in the rural areas. As noted above, the agro-food sector attracts migrant workers who hop from one seasonal job to the next. For individual workers, it is not easy to search for available farmwork jobs; even when they find a job, it is difficult to organize transportation or accommodation (farms may be far from a village or town centre). On the part of farmers or plantation owners, too, finding the right number of workers, who will arrive at the right time and stay for the necessary amount of time, is not easy. And again, accommodation and transportation need to be organized. For these reasons, migrant farmwork has historically been organized through layers of informal, and even illegal, labour brokers: from those who recruit workers from their home villages in their home countries, to those who act as team leaders at the work site, to the principal labour contractor who signs a contract with the farm owner and is the direct employer of the migrant workers. S/he usually sets up the workers’ accommodation, and organizes the daily transportation from the camp to the work site, moving workers from one farm to the next.

Many migrants work illegally, in the sense that they stay and work in the country of the job site without the necessary legal permits. It may appear that the authorities are shockingly ignorant of the presence of migrant workers concentrated in places of work or camps. However, given the need for migrant workers at the work sites, the authorities generally turn a blind eye to the violation of rules and laws, except when occasional waves of anti-migrant sentiments force them to take action. The history of migrant farmworkers in California from the 1850s, mentioned earlier, is a classic example of this contradiction: workers who are essential but at the same time illegal (Holmes 2013; McWilliams 2000 [orig. 1935]; Mitchell 2012; Ngai 2014 [orig. 2004]).

Informality and illegality ensure the relative cheapness of migrant labour power: owners of capital and labour brokers do not have to pay migrant workers the legally mandated minimum wage, they do not have to pay for periods when the workers are not needed, and they do not have to pay part of the cost of social reproduction—healthcare, childcare, social insurance, pension, paid holidays, paid sick leave, maternity and paternity leave, overtime and working holiday wage differentials, and so on (Burawoy 1976). From recruitment of prospective migrant workers, to their transport across the border, and into their possible work sites; from finding work, to getting organized in carrying out jobs; to going back to Myanmar during normal times or emergency situations—all these social processes are carried out by migrant workers by embedding themselves within complex social relations dominated by labour brokers and entrepreneurs: moneylenders, merchants, scammers, swindlers, petty entrepreneurs relying on transactions with migrants.

Our research suggests that, based on the living standards of the countries of destination of migrant workers, especially China, Thailand, and Malaysia, the wages paid to Myanmar migrant workers are—*at best*—at the mandated minimum wage levels of those other societies. It is important to remember that these wages are paid only for short, seasonal periods, with no payment during work gaps, e.g., between sugarcane cutting seasons. Moreover, based on interviews for this study, this wage (usually aggregated and averaged in monthly terms for comparison across countries) is earned by working non-stop for 7 days a week (*not* 5 days a week) and in some jobs, such as sugarcane cutting, by working long hours each day because the wage system is usually piece rate. In addition, migrant workers have to pay for the cost of securing the job: fees for labour brokers, transportation to and from the job site, cost of renewing their permit to stay (if their stay is legal). Further, there are a number of critical issues linked to this. First, there is a significant difference between the minimum wage standard in Myanmar and in the countries of work, in which it is much lower in the former. Second, the legally mandated minimum wage levels in these countries are low relative to the standard of living in their own contexts. Third, while workers who follow legal and formal migrant work channels (usually in the form of a short-term ‘permit to stay’, at least in the case of China) are not automatically guaranteed higher wages and better living conditions, they almost always enjoy relatively better processes and outcomes in terms of wages and working conditions than their counterparts who use illegal channels and do not have proper documents. Fourth, the kind of social and health insurances and other workers’ rights and benefits that one would expect in regular employment are virtually unheard of in the world of migrant workers, especially those in the agro-food sector.^20^

In Yunnan—the destination of most China-bound Myanmar migrant workers—the minimum wages in townships, counties and small cities outside the provincial capital range from US$200 to US$250 per month as of May 2020. These rates are low compared to the minimum wage levels in key Chinese cities like Shanghai (US$380 per month). This means that, increasingly, Chinese labour prefers to work in key cities and provinces with higher minimum wages, creating the condition for cheap labour from Myanmar to expand rapidly in agricultural areas and in small to medium towns and cities in Yunnan. Many of these rural villages are half empty because of massive rural–urban migration in these counties (Murphy 2002; Ye 2018), and thus faced labour shortages when crop booms (sugarcane, eucalyptus, etc.) started, especially in southern China (Borras et al. 2018; Xu 2019). But even the low minimum wage levels in the townships and counties of poorer provinces in China are much higher than the mandated minimum wage in Myanmar which was set in 2019 at US$3.45/day, equating to approximately US$82/month for 24 working days—just about a third of the lowest possible minimum wage levels for farmwork in Yunnan. Based on our interviews, a migrant worker earns around US$215/month in farmwork, especially for sugarcane cutting, and doing all sorts of odd jobs in townships and small cities in Yunnan. This is just about the minimum wage for those areas in Yunnan (without the non-wage benefits legally mandated for formal jobs). Basic food and accommodation are almost always supplied ‘free of charge’ by the labour contractor, at least in farmwork in China (or rather, their real cost is already tucked in the wage structure).

In comparative glances, as of 2018, the minimum wages in Thailand, depending on the regions, are from 313 Baht to 336 Bath (US$10.3 to 11/day).^21^ If they work on the lower level of this scale, and work for 24 days in a month at 8 h/day, they can earn up to 350,000 Kyats (US$272), as long as they receive the full minimum wage, and do not have to spend too much for accommodation and food expenses, and for labour brokers. In Malaysia in 2019, the minimum wage was RM1,100 (US$267/month) and was supposed to increase in 2020 to RM1,200 per month (of 24 working days).^22^

Many people we interviewed stressed two points, namely, first, they can potentially earn twice or three times as much working abroad as they could working inside Myanmar, and second, when it becomes possible (even during the pandemic) they intend to go back to China or Thailand or Malaysia to work. Such eagerness to return does not necessarily mean they do not have serious problems at their work sites. Even before the pandemic, not every migrant worker was lucky enough to receive a minimum wage or to enjoy good working conditions, as suggested by a 2017 ILO report by Harkins and Ahlberg on access to justice with specific reference to migrant workers from Southeast Asia (Myanmar, Laos, Cambodia, Vietnam) working in the region (see Harkins and Ahlberg 2017, Figs. 5 and 6, pp. 25–26). Non-payment and underpayment of wages were among the most prevalent problems faced by migrant workers, including those from Myanmar, in China, Thailand and Malaysia. In this ILO report, the most common problems reported by migrant workers in the region were: withholding of documents, wages below the legal minimum, no work leave, excessive work hours, and contract substitution/changes. The ILO study suggests that paying below the minimum wage standard or by withholding wages and other problems flagged in our interviews were actually not unique to the pandemic era, and seems to be routine in the world of migrant labour—that are likely to have been exacerbated by the pandemic.

In short, informality and illegality play a critical role in facilitating the insertion of migrant workers into the labour regime. The terms of their incorporation are not static or uncontested. Nevertheless, it is rare to see organized political contention by migrant workers, at least in the sites of our study. The coercive mechanisms deployed to ensure informality and illegality keep migrant workers generally unorganized and politically weak. O’Laughlin (1977, p. 29) emphasizes that “the conditions which underlie the relative powerlessness of certain workers are in any circumstances political as well as technical, and cannot be understood simply in terms of the functional requirement of capital”. She concludes: “People don’t accept low wages just because they can go home to eat”.

## The continuing process of generating migrant workers

I live in an IDP camp. I have no farmland in the camp. No one has farmland in the camp. But I have farmland in my original village. We planted paddy and corn, and raised chickens and pigs in our compound there. But then there was fighting between the military and ethnic armed groups. So, we had to leave, and ended up in this camp. I studied at the Lashio Teachers Training College, and accumulated debts because of that. That is why I went to work in China to pay off my debts. I earned US$230–290/month before the pandemic. But during the pandemic, there were a lot of restrictions on wage work, and I earned only US$100–120/month. That’s why I decided to go home in May 2020.

Poor peasants and pastoralists are socially differentiated through market relations or expelled from their lands via extra-economic coercion. Brute force and violent conflict are directly associated with many extra-economic coercion, such as violent land grabs, deployed by the militaristic central state. But even those spared from direct violence, market relations are, to varying degrees, shaped and mediated by the culture and practice of violent conflict. This is partly how the rural working class has emerged as a class, and this is how it is being maintained as a social category. Many, but not all, of them have been completely divorced from their means of production. Boutry et al. (2017) found out that in their surveyed villages in the Dry Zone, 41% of households are completely landless (in terms of farmland). Although this also means that the majority still have some access to some farmland, the same study identified a trend in distress farmland sales and renting out of farmland by plot holders. Against this background, we can speculate that the pandemic could lead to widespread distress farmland sales and renting out of peasant and pastoralist farm and pasturelands, or to a process of going back to work the land with greater intensity in order to earn a living, in the absence of wage work. A combination of the two could mean selling or renting out a portion of the farmland, while intensively working the remaining portion. Whether such processes occur, and to what extent, are empirical questions that need to be investigated.

A significant portion of those who end up as migrant workers are those who lost farmland, or whose parents lost farmland, because of the cycles of land grabbing over the past two recent decades, or younger people whose villages lost their traditional ‘community farmland reserve’ through land grabbing.^23^ This happens in a number of ways. There are communities in Myanmar that saw militarization of various forms, scale, and intensity, often in relation to the effort of the Myanmar military (Tatmadaw) to fight ethnic armed groups and extend the territorial claim of the Myanmar central state. This has led to people fleeing from their villages in waves during the past decades. Powerful actors, including the central state, military, owners of capital, loggers, mine prospectors, big conservation organizations, or militia, in turn, have taken over many of the lands abandoned by the villagers, while most of the latter end up becoming IDPs (Borras et al. 2020a, b; Franco and Borras 2019; Woods 2011). As of February 2021, the estimate by UNHCR (The UN Refugee Agency) of the total IDP and so-called ‘stateless persons’ (i.e., Rohingya) was placed at 0.77 million, which was slightly lower than the 2017 data of 0.85 million.^24^ It is likely to be an underestimation. Counting IDPs is not straightforward, given the fluidity of their situation, but it is likely that many IDPs become migrant workers. The reality is that the social processes that generate IDPs have not stopped: militarization has continued (Kramer 2020). This generator of cheap migrant labour churns incessantly, assuring owners of capital in Myanmar and abroad of an endless supply of cheap labour.

Further, there are coercive legal measures that facilitate the separation of rural villagers from their means of production and social reproduction, especially land. This is case of the Vacant, Fallow and Virgin (VFV) Land Law which was passed in 2012. The scheme is simple: villagers are asked to register their lands, supposedly to lead to a formalization process and thus to formal land tenure. By the same legal procedure, all land that is not claimed, cannot be claimed, or is unsuccessfully claimed by villagers becomes ‘free land’ for the state to award to corporations or big conservation projects under various forms of land concessions. There are many reasons why villagers might not be able to register: they are engaged in shifting agriculture which is not formally allowed; they are not aware of the law; they are not aware of the procedure; they do not have the logistical means to pursue their claims; the counter-claims of competing elites are stronger and more successful; or a combination of these factors. The VFV Land Law is not the only mechanism for grabbing land from the villagers, but it is a key one. By early 2013, almost 2 million ha of land (farmland, wetlands, and forested lands) had been given to various concessions (Thein et al. 2018; Myanmar Union Government 2016).

## Toward a new old normal? Concluding discussion and implications

Talk about a ‘new normal’ in a post-pandemic world abounds. What does a ‘new normal’ mean for rurally rooted cross-border migrant workers? The issue of the future of migrant workers when they are no longer physically able to work, including the issue of eventual retirement, has been an uncertain and complicated matter for them; it has become even more so because of the pandemic. Migrants’ home villages are among the places where state-provided health care and social security support barely exist, and so they themselves are shouldering the key elements of social reproduction. Rooted in the countryside, left-behind families’ short-term to long-term survival (including labour recuperation and labour retirement) thus depends hugely on the socio-economic conditions prevailing in their home communities. But in too many instances the socioeconomic health of rural villages is critical and the prognosis is poor. Even for migrants who earned some kind of pension, which is rare, life remains hard, as can be seen in the two lives of two interviewees in our study.

I have been a farmer since 1990, inheriting land from my parents for paddy production. But farm income was not enough, and we accumulated debts. In 1999, my husband and I went to Thailand. We paid a lot of money to the labour broker. I started working at a vegetable farm. After two years, I was able to pay off the debt to the broker, but not the debts back home, and the interest kept growing. I moved to work at a plastic factory, then later at a cotton blanket making factory for 15 years. I was asked to retire by the factory as I turned to the retirement age. With pension money and ready to retire we went home to Myanmar in March [2020] just as Covid-19 was starting. Despite all the earnings all these years and the pension money, we were left only with a small amount of money and around 16 grams of gold. We were able to support our children’s education expenses and helped open a motorbike repair shop for our eldest son. But our son earns just enough for him to survive. Our second son is still very young. We own about 5 acres of farmland. We prefer to just spend our time doing religious devotion. But we have to worry for the family survival and for the younger son’s education. But my husband and I started to suffer from chronic back pain. We have no other livelihood options, and so we can only do farming. But if during this year we won’t have a good amount of harvest, we won’t farm again. We would just rent out the land to others. People who can do farming in large area of land come and rent the farmland. We don’t know what the livelihood opportunities are for us next year. We want our second son to quit school and go to Thailand, but we also pity on him. If the gate from the Thai side will be open, and if he wants to go, we would let him. To survive becomes the most important aspect for us. We have to generate income from whatever means available.^25^

I worked as a sugarcane cutter in China. In the beginning, the boss provided us the meals but later on there were many illegal migrant workers from Myanmar, and he started not to provide any meals to workers anymore. The boss paid daily wages to us. We had to spend on food costs, using our daily wages. They didn’t provide a place to stay, so we just made temporary tents in the sugarcane field and stayed there. Not only us, but all migrant workers who came and worked had to make a tent and stayed in the field. We went home because of the pandemic. But there are no regular jobs in or near our village. It’s difficult for us to survive here. My parents are poor and my family doesn’t own land. We rent land from others and grew paddy on *taungya* before, but it’s very low yield. We can’t afford to buy farmland. Therefore, I have to migrate again so that my family will survive. I will leave my children with my mother, and my husband and I have to go to China again after this pandemic.^26^

**Fig. 3:**
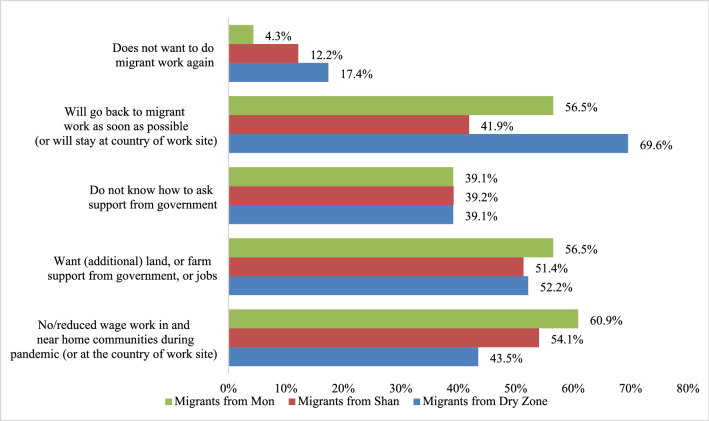
Migrant workers’ perspectives on current condition and plan for a post-pandemic period (% of migrant workers interviewed in 2020, N = 120: Dry Zone: 23; Shan State: 74; Mon State: 23)

Both interviewees think and worry about their future, whether one is referring to a retirement or just starting a young family. Belonging to a community and thus, having some kind of access to land or space in the home village is key for both the interviewees: a place to retire, and a space in the home village for extended family members who could take care of the left-behind children. There is a pattern among migrants’ responses to our interviews (see Fig. 3). First, 43%, 64% and 61% of Dry Zone, Shan and Mon migrants, respectively, said there is no wage work or reduced wage work in or near home communities during the pandemic (or in the country of the work site for those who opted to stay). Second, 51%, 52% and 56% of Shan, Dry Zone and Mon migrants, respectively, said that they want farmland, or additional farmland, and/or farm support, and/or jobs from the government in or near their home villages; if this were forthcoming, they would seriously reconsider doing migrant work again. Third, 42%, 56%, and 70% of Shan, Mon and Dry Zone migrants, respectively, said they will go back and do migrant work abroad again as soon as it is possible to travel and work; for those who decided to stay in the countries of their migrant work, they will stay there to keep their job, or look for new jobs. Only a small percentage said they do not want to do migrant wage work again, and some of these were already at the retirement stage, after having worked for many years as migrants.^27^

By way of conclusion, we would like to highlight four overlapping points that help us to contextualize rurally rooted cross-border migrant workers, the Covid-19 pandemic, and a just transition into a future that is fairer and kinder to working people. Our starting assumption is that it is possible to explore such a positive future only by situating migrant workers within a unitary view of production and social reproduction that has been impacted by the pandemic.

First, the basis for the ‘rural-rootedness’—past, present, and future—of this stratum of working people lies centrally in the strategic location of land in the spheres of production *and* social reproduction: to produce or access things of use value and exchange value, not only to reproduce labour power, but to reproduce one’s self as a human being more generally. The value of land is not just to be able to cultivate produce or raise animals for use and/or exchange value, but to be able to *gather*, *harvest*, or *forage* things from the land, water, or forest. Land access for housing, space for kitchen and medicinal gardens, and a tiny space for livestock are key for working people (Pritchard et al. 2019; Rammohan et al. 2019). *Land access facilitates access* to other resources, for example springwater for drinking, cooking, or washing, which may not be accessible if a spring is privately enclosed. Land access is key to *enabling* key social reproduction tasks, such as a plot of land for a community day-care centre, a health clinic, or a multipurpose community centre for social gatherings. Land access has a value for the *socialization*, *leisure*, or *nostalgia* that it enables through land-dependent social activities and institutions such as community parks and playgrounds; places for worship like a church, a pagoda, or a dwelling for spirits; or a cemetery. Land access conjures and concretizes a place to satisfy one’s feeling and sense of *longing and belonging*. Land access *allows* for the reproduction of people as a distinct social group based on race or ethnicity. This range of access, in many rural communities, has a dynamic temporal aspect: future households are assured of resource access through community land reserves. This represents a range of meanings and types of value of land (Franco et al. 2015), and it is useful to analytically cluster them into productive and social reproductive spheres. This *range of access to a range of land* and resources inherently entails a range of institutional mechanisms, perhaps broadly categorized between private and individual on the one hand, and community-based and socialized on the other hand, allowing for an infinite number of possible hybrid combinations. Thus, the dominant terms ‘landless’, that almost always means those without farmland, and ‘landholder’ (small landholder, landholder-worker) —in the context of our study—muddle rather than clarify the analysis and need to be recast. The *dissolution*, *diminution,* or *degradation* (all inherently a matter of degree) of this range of access to a range of land and resources alter the very conditions for production and social reproduction of working people.

Second, the politics of land (who gets what type, degree, and quality of access to which types of land, how, and for what purposes) shapes a labour regime; conversely, a potential or actual labour regime shapes the politics of land. Broadly cast, land plays a central role in the co-constitutive spheres of production and social reproduction. A view that equates land only with economic production, that is, a plot of farmland, belongs to the perspective that privileges the sphere of production over social reproduction. Building from Shah and Lerche (2020), Bhattacharya (2017), Fraser (2016), and O’Laughlin (1977), we argue and conclude that ‘land access’ has to be reframed into something broader that covers productive *and* social reproductive spheres. Land access has to be disentangled from an overly production-centred treatment, and shifted instead to a production/social reproduction-centred perspective.

Third, having a range of access to a variety of land and resources shapes the complex of social reproduction which—as Fraser, Shah and Lerche, and others, argue—conditions the possibilities for productive work. Our study highlights a tendency among migrant workers to rely primarily on kinship ties, and secondarily on the local community, to look after small children and the elderly while both parents do migrant work. For either the parents or their relatives to have a house of their own in the village (whether on a freehold property or on a village commons) is key to this social reproduction imperative. However, this is not the only possible course of action: there are increasing instances of parents bringing their babies and small children to their migrant work sites, either because there are no appropriate kin to look after the young ones, or because parents are too emotionally distraught at being away from their infants. The options for, and decisions concerning, productive work hinge on the worker’s social reproduction imperatives; conversely, their social reproduction circumstances shape their options and decisions with regard to productive work.

Fourth, as described by our study, market relations and violent coercion led villagers to become situated in an extremely precarious social class category and led them to pursue cross-border migrant work. The long history of violence deployed by the militaristic Bamar central state against working people played a key role in the construction and maintenance of the latter as a social class. Whether or not the working people will be able to gain and maintain a range of access to a range of land for productive and social reproductive activities depends largely on whether or not the mighty state apparatus of violence and coercion would stop being used against them. This fundamental question has become even more so in light of the violent military coup of February 2021 (Ra et al. 2021).

Fifth, a unitary view of production and social reproduction in the context of rurally rooted migrant workers requires a similarly unitary view of land and labour, and labour justice and agrarian justice issues. This has implications for organizing and mobilizing of migrant workers. If a positive post-pandemic future is to be constructed, rurally rooted cross-border migrant workers will have to be part of the process of imagining and constructing such a future. Organizing and mobilizing migrant workers in the agro-food sector is possible, as history has shown (e.g., McWilliams 2000 [orig. 1935])—but it is rare. The fragmentation of the classes of labour (Bernstein 2006), especially in the era of neoliberal capitalism, has weakened conventional trade union struggles. Shah and Lerche (2020, p. 728) argue that there are many obstacles to organizing and mobilizing migrant workers: “Language barriers, treatment of migrants as second-class citizens, and permanent transience lead to isolation… Circulation in work with no protection can encourage low-caste labourers to be dependent on political patronage and persistent debt-bondage.” They conclude: “How can people organise if one year they are in one site and in another year in an entirely different place?… The working class movement itself can also be a barrier. It has neglected migrant workers…” (ibid., pp. 728–729). If we look at political struggles from a starting point of, but not limited to, the agrarian end of the continuum, it is possible to add a layer of political complications to what Shah and Lerche have outlined. It might be useful to think in terms of geographic and political sites of organizing and mobilizing not as a question of either factories or fields, dormitories or homes, land or labour, class or community; rather, it is all of the above, simultaneously. But how actually to organize and mobilize in such a continuum is easier plotted in an academic environment than implemented in the trenches.^28^ This increases the political challenges both for worker trade unions and for agrarian movements.

Returning to where we started in this paper, namely, the importance of rural and migrant workers on the one hand, and the peasantry and alternatives such as food sovereignty and agroecology in a post-pandemic future on the other hand, we explore some possible implications of our study for political struggles, speaking more directly to agrarian movements.

One implication is that constructing a peasant- or small-scale farmer-centric vision of a post-pandemic future based on concepts and political projects such as food sovereignty, without confronting relevant class and power relations within the community and the household, between family farmers and rural workers (migratory wage workers or not), between men and women, older and younger members of a household, all entangled in the same agro-food system, is inherently problematical. The last three decades have seen a surge in agrarian movements worldwide that recast the agenda of public discourse on land, food, environment and climate change, human rights, seed technology, and agroecology (Desmarais 2007; Edelman and Borras 2016; Holt-Giménez and Shattuck 2011; Martinez-Torres and Rosset 2010; Wittman et al. 2010). The most consistent and significant blind spot in the agenda of agrarian and food movements is labour.

We understand labour in two political senses. First, as the implications of class relations wherein farmers (especially surplus-producing capitalist farmers, but not only) and other key food-system social groups generally of the petty bourgeoisie (food processors, food store owners, food distributors, restaurant owners, etc.) occasionally or regularly hire workers (farmworkers, cooks, service delivery personnel, etc., migrant or otherwise). Second, as the implications of the fact that a significant section of the world’s peasants occasionally or regularly hire out labour themselves (both as a matter of degree), conforming less to the conventional peasant or farmer label, but rather approximating the description of ‘working people’ (Shivji 2017) or ‘classes of labour’ (Bernstein 2006). If we take the issue of labour in the two senses described here—aware of the fact that it is likely to expose latent class contradictions and political tensions among the objectively existing ‘people of the land’, to varying degrees (Bernstein 2014)—the question arises: is it necessary and possible to rethink and reframe food sovereignty or agroecology from the perspective of ‘working people’ or ‘classes of labour’, and if so, how?^29^ It is easier to claim that farmers will take up the issue of migrant workers than to operationalize it, to address it in scattered and isolated local instances, than to institutionalize it class struggle- and system-wide. Moreover, central to food sovereignty is the issue of reversing the ‘distancing’ in the global industrial food system; thus, the ‘localization’ component of food sovereignty is key: to produce food in and near one’s territory (Robbins 2015). The situation of cross-border migrant workers is the opposite: it is defined by ‘distancing’, and this is where the challenge is. How are we supposed to imagine the rural working people (the peasants who are migrant wage workers, or migrant wage workers abroad who are at the same time peasants in their home community) getting involved in the construction of food sovereignty—whether we think of the latter in the migrant work site abroad or in their home community? The propositions for a post-pandemic future based on food sovereignty and agroecology (Altieri and Nicholls 2020; Montenegro de Wit 2021; van der Ploeg 2020) can be made stronger, conceptually and politically, by confronting the issues raised in relation to labour.

The second implication is that our study shows the need to consider land more systematically from the perspective of its location in production and social reproduction (see also Pattenden 2018). Contemporary land struggles cannot be reduced to the question of allocating a peasant household an economically viable farm plot. A replotting of land demand based on actually existing sets of productive and reproductive activities of peasants—or, indeed, working people—will be needed urgently, veering away from either a production-centric or a social reproduction-centric perspective. In contrast to the conventional land mapping that usually plucks out a piece of farmland and a house plot from their broader social, ecological, and community embeddedness and entanglement, what is likely to emerge in such an alternative exercise is a ‘land–labour map’ shaped by mutually constitutive relations of class, ethnicity, gender, and generation (Bhattacharya 2017; White 2020) that is akin to a web: farm plot; house lot with food garden; access to common forest, grazing land, parks and playgrounds; plots for day-care centre and school, and places of worship; access to a landing spot when river, lake, or sea fishing is a complementary livelihood. A range of access to such diverse and multiple lands—rather than access to one or two specific plots, such as a fixed area of farmland—helps ensure that the question of land is not artificially divorced from labour, and that land and labour are considered simultaneously in production and social reproduction.

Recent explorations on agrarian change and social reproduction by Cousins et al. (2018), Jacobs (2018), and Pattenden (2018), among others, offer useful and important signals. As Levien et al. (2018, p. 876) argue: “Struggles over means of both production and social reproduction remain as important as ever, but are not playing out in remotely the same way as Marx predicted. Land remains an important focus of such struggles, even if its precise significance remains fiercely debated”. This speaks to the formulation by Bernstein about land struggles in relation to classes of labour, with his starting point that, “struggles over land may manifest an agrarian question of (increasingly fragmented) classes of labour, but—for all their importance—do not have the same systemic (or world-historical) significance as the agrarian question of capital once had” (Bernstein 2006, p. 449). He further explains: “this is not to deny the class impulses underlying struggles for land… Nor is it to withdraw political sympathy and support for such struggles because they fail to satisfy the demands of an idealized (class-purist or other) model of political action” (ibid., p. 459). He concludes with the need to recognize and analyse “the contradictory sources and impulses—and typically multi-class character—of such struggles, in ways that can inform a realistic and politically responsible assessment of them” (ibid., p. 459).

The dominant framing in agrarian and food movements’ claims about political struggles is that land struggles are urgent and necessary in order to pursue a positive future via the ‘peasant way’, whether this is about the broader food system (food sovereignty) or farm systems (agroecology). In itself such a claim needs careful empirical investigation, at least in the limited context of rurally rooted cross-border migrant workers, because it is probable that most of the working people in this group may not want to see themselves cast in the idealized image of a full-time peasant (this is speculative, and warrants further investigation). It is also compelling to have a careful empirical investigation regarding actual demand and political struggles for land—as broadly framed in our study within the unitary sphere of production and social reproduction—whether these are captured by agrarian and food movement narratives, and if so, to what extent. Our guess is that the former is not fully captured by and represented in movements’ masterframes—at least not in the emerging agrarian social movements and networks in Myanmar, as can be deduced, for example, from Ra and Ju (2021) and Sekine (2021). It is necessary and possible to reframe political struggles for land from the perspective of working people or classes of labour, which requires struggles to be detached from a ‘small family farm-centric’ and/or from an ‘economic production-centric’ masterframe, to a ‘working people-centered’ and ‘production/social reproduction-centric’ perspective. Treating production and social reproduction as a single unit of inquiry and political intervention in the context of rurally rooted cross-border migrant workers requires an inherently interlinked view of labour, agrarian, and food justice struggles, informed by class, gender, generational, and ethnic perspectives. But forging coalitional politics among working people against external capital, while not losing sight of the exploitative relations within their communities and the household, will be challenging. It will require a messy, recursive process of theoretical exploration and practical politics. We locate our paper in this difficult but necessary conversation and political process.

## Annex 1: Profile of interviewees

**Table:**

Time	Items		Migrants from Dry Zone	Migrants from Shan State	Migrants from Mon State	Total
2020 (during the pandemic)	Number of interviews		23	74	23	120
	Gender	Male	18	43	14	75
		% of the region	78.3	58.1	60.9	62.5
		Female	5	31	9	45
		% in the region	21.7	41.9	39.1	37.5
	Age	< 25	7	43	5	55
		% of the region	30.4	58.1	21.7	45.8
		26–49	15	30	15	60
		% of the region	65.2	40.5	65.2	50.0
		> 50	1	1	3	5
		% of the region	4.3	1.4	13.0	4.2
	Land access	With farmland	14	19	7	40
		% of the region	60.9	25.7	30.4	33.3
		Without farmland	9	47	13	69
		% of the region	39.1	63.5	56.5	57.5
		No Information	0	8	3	11
		% of the region	0.0	10.8	13.0	9.2
	Work site	China	16	49	0	65
		% of the region	69.6	66.2	0.0	54.2
		Thailand	4	22	21	47
		% of the region	17.4	29.7	91.3	39.2
		Malaysia	3	1	2	6
		% of the region	13.0	1.4	8.7	5.0
		Other	0	2	0	2
		% of the region	0.0	2.7	0.0	1.7
	Response to the pandemic	Return home	18	62	17	97
		% of the region	78.3	83.8	73.9	80.8
		Remained in the countries where they work	5	12	6	23
		% of the region	21.7	16.2	26.1	19.2
August–September 2019	Number of interviews		16	0	0	16
	Gender	Male	14	0	0	14
		% of the region	87.5	–	–	87.5
		Female	2	0	0	2
		% of the region	12.5	–	–	12.5
	Age	< 25	9	0	0	9
		% of the region	56.3	–	–	56.3
		26–49	7	0	0	7
		% of the region	43.8	–	–	43.8
		> 50	0	0	0	0
		% of the region	0.0	–	–	0.0
	Land access	With land	4	0	0	4
		% of the region	25.0	–	–	25.0
		Without land	3	0	0	3
		% of the region	18.8	–	–	18.8
		No information	9	0	0	9
		% of the region	56.3	–	–	56.3
	Work site	China	16	0	0	16
		% of the region	100.0	–	–	100.0

## Footnotes

1 https://www.eurovia.org/open-letter-on-urgent-and-necessary-measures-for-rural-workers-in-the-context-of-covid-19/.

2 https://viacampesina.org/en/the-winds-of-change-are-blowing-harder-covid-19-update-on-peasants-rural-workers-and-other-marginalized-groups/.

3 This paper draws from Borras et al. (2020a, b).

4 We use the term ‘peasants’ here guided by Edelman's (2013) definitional discussion.

5 A critical background to the argument for a unitary view is explained by Fraser (2016, p. 99), and warrants an extended quote: “Historically… processes of ‘social reproduction’ have been cast as women’s work, although men have always done some of it too. Comprising both affective and material labour, and often performed without pay, it is indispensable to society.” She concludes: “Without it there could be no culture, no economy, no political organization. No society that systematically undermines social reproduction can endure for long. Today, however, a new form of capitalist society is doing just that.” Fraser elaborates: “The result is a major crisis… of social reproduction… The social reproduction strand forms an important dimension of this general crisis but is often neglected in current discussions, which focus chiefly on economic or ecological dangers.” She argues that, “This ‘critical separatism’ is problematic; the social strand is so central to the broader crisis that none of the others can be properly understood in abstraction from it.” And concludes: “However, the converse is also true. The crisis of social reproduction is not freestanding and cannot be adequately grasped on its own.”.

6 Fraser also explains that, “Other background conditions include the governance functions performed by public powers and the availability of nature as a source of ‘productive inputs’ and a ‘sink’ for production’s waste” (2016, p. 101). In this paper, we use ‘land’ as a loose shorthand for ‘nature’, and we view it in the context of social reproduction not just as a source of productive inputs and a ‘sink’ for production’s waste; rather, in the context of rurally rooted cross-border migrant workers, land/nature is indispensable for a range of social reproduction activities that are not always systematically addressed in the literature, at least not in the land literature.

7 We started the research in the Dry Zone before the pandemic. This region is predominantly Bamar region, marked by a dry agroecological condition hit by recurring drought. Lack of irrigation is a very common issue among villagers. The areas near major urban centers have seen widespread cases of land grabbing during the past two or three decades. The two ethnic borderland states, Shan and Mon, have been key sites of long-drawn violent confrontation between the Burmese army of the central state and the various ethnic armed groups in these two states, resulting in waves of internally displaced peoples (IDPs), many of whom lived in IDP camps, and even more IDPs ended up seeking migrant wage work inside and outside Myanmar. Customary land tenure and production systems have persisted, although waves of land grabbing during the past two decades or so have been progressively eroding such customary system. Most Mon migrants go to Thailand, many of those from the Shan State seek migrant work in China, while many of those from the Dry Zone in recent years tend to go to China (see Fig. 1, map; see also Deshingkar et al. 2019). We wish we could make some comparative analysis across the Dry Zone, Shan and Mon States. Unfortunately, we did not construct our data gathering method for such a comparative study. But we will keep this in mind for our future, follow up research undertaking.

8 Additionally, some of the co-authors conducted fieldwork in Yunnan in November–December 2019, and interviewed sugarcane cutters and labour contractors. While data from that field is not directly used in this paper, it has helped us understand better the context on the side of China. But what our method implies is that our dataset is not representative of Myanmar-wide situation, and thereby analytical exploration towards generalization based on our dataset may not be possible. Yet, the insights that our limited dataset provides is important, and should serve as basis for a more robust scientific research on relevant research agendas in the future.

9 Telephone interview with a 30-year-old man from Sagaing State, May 2020.

10 Telephone interview with a 28-year-old woman from Mon State, May 2020.

11 Telephone interview with a 20-year-old man from Mon State, May 2020.

12 https://www.iom.int/countries/myanmar; downloaded on 29 May 2020.

13 World Bank Databank: https://databank.worldbank.org/views/reports/reportwidget.aspx?Report_Name=CountryProfile&Id=b450fd57&tbar=y&dd=y&inf=n&zm=n&country=MMR; downloaded 29 May 2020.

14 Asian Development Bank (ADB), Key Indicators for Asia and the Pacific 2020, https://kidb.adb.org/; downloaded 11 May 2021.

15 This is a common view among those we interviewed.

16 Face-to-face interview, Magwe, September 2019.

17 The interviewers among our study team members were not consistent in terms of follow-up questions on this matter, namely, about the variables that are relevant to farmland production and income generation, including prices of produces, costs of inputs, irrigation, and so on. On these issues, therefore, our dataset does not allow us to claim any insights with confidence.

18 Face-to-face interview, September 2019, Bagan.

19 Based on various interviews (with labour contractor, farm workers, and county officials) done by three of the co-authors during their field work in Yunnan province in November–December 2019.

20 Farmwork is notorious for this, but even those based in urban centres, such as the large numbers employed in the food service/hospitality sector (e.g. restaurant workers) are in a similar position, as Anthony Bourdain revealed two decades ago (Bourdain 2000).

21 https://www.nationthailand.com/news/30379141; downloaded 02 June 2020.

22 https://www.thestar.com.my/news/nation/2020/01/14/new-minimum-wage-gazetted; downloaded 02 June 2020.

23 In our study, those who do not have access to farmland now are: (a) young people many of whom have parents who have farmlands, (b) Internally Displaced Peoples (IDPs), many of whom live in IDP camps; all those interviewed said they have farmland in the original village, but not since they become IDPs; and (c) others who are old enough to have farmland but for various reasons – aside from the two mentioned above – have no farmland. If the situation in Myanmar would follow the global trend, only a handful of the peasants’ sons and daughters of today would manage to secure farm from their parents at some point in the future, and usually only smaller and fragmented plots (White 2020). Theoretically, the legal-political remedy for the current landlessness of IDPs is land restitution. But we have yet to see a truly successful land restitution in any country of the world in contemporary times (see Mark and Belton 2020). For the third category of the landless and land-poor, the historically proven effective legal-political policy instrument is redistributive land reform, a difficult but not impossible approach to address landlessness. All these categories are core components of contemporary Myanmar rural working people, many of whom resort to alternative or complementrary cross-border migrant wage work.

24 https://reporting.unhcr.org/myanmar. Downloaded 14 February 2021.

25 Telephone interview, May 2020, retired 57-year-old woman from Mon State.

26 Telephone interview with a 25-year-old Kachin woman from Shan State, May 2020.

27 Unfortunately, we did not include questions related to whether there are differences between those with farmland and those without farmland in terms of what kinds of migrant work they do and the terms and conditions of their migrant work.

28 Myanmar has a relatively vibrant emerging trade union movement. Labour unions have been relatively active in union struggles around wage, benefits and working conditions. The strength of the unions is concentrated in the large garment sector (see Arnold and Campbell 2017 for analysis of the evolving labour regime and union organizing in Myanmar). Some Myanmar workers based in Thailand or Malaysia have intermittently raised worker issues there, although not for the ones in China. he unions were among the first ones to have protested against the February 2021 military coup. In response, the military junta quickly illegalized the key autonomous unions, and union activists have been arrested or went into hiding. As of this writing (June 2021), except for those based abroad, labour unions have been silent. Workers are struggling to hold on to whatever jobs they have, which is becoming harder after the coup. Many factories have been shut down. Given the evolving character of autonomous union movement in Myanmar, and adverse impact of the pandemic and the military coup on them, we think that serious considerations on some of the key points that we described in this study might contribute to possible adjustments in their political organizing and mobilizations strategies.

29 Bernstein is also sceptical as to whether a peasant-based food sovereignty can feed the world’s working class who need food but do not produce food (Bernstein 2014).
